# Combined effects of double mutations on catalytic activity and structural stability contribute to clinical manifestations of glucose-6-phosphate dehydrogenase deficiency

**DOI:** 10.1038/s41598-021-03800-z

**Published:** 2021-12-21

**Authors:** Phonchanan Pakparnich, Sirapapha Sudsumrit, Mallika Imwong, Teeraporn Suteewong, Kamonwan Chamchoy, Danaya Pakotiprapha, Ubolsree Leartsakulpanich, Usa Boonyuen

**Affiliations:** 1 Department of Molecular Tropical Medicine and Genetics, Faculty of Tropical Medicine, Mahidol University, Bangkok, 10400 Thailand; 2 Department of Chemical Engineering, School of Engineering, King Mongkut’s Institute of Technology Ladkrabang, Bangkok, 10520 Thailand; 3 Princess Srisavangavadhana College of Medicine, Chulabhorn Royal Academy, Bangkok, 10210 Thailand; 4 Department of Biochemistry, Faculty of Science, Mahidol University, Bangkok, 10400 Thailand; 5 Center for Excellence in Protein and Enzyme Technology, Faculty of Science, Mahidol University, Bangkok, 10400 Thailand; 6 National Center for Genetic Engineering and Biotechnology, National Science and Technology Development Agency, Pathum Thani, 12120 Thailand

**Keywords:** Biochemistry, Biophysics, Molecular biology, Molecular medicine

## Abstract

Glucose-6-phosphate dehydrogenase (G6PD) deficiency is the most common enzymopathy in humans, affecting ~ 500 million worldwide. A detailed study of the structural stability and catalytic activity of G6PD variants is required to understand how different mutations cause varying degrees of enzyme deficiency, reflecting the response of G6PD variants to oxidative stress. Furthermore, for G6PD double variants, investigating how two mutations jointly cause severe enzyme deficiency is important. Here, we characterized the functional and structural properties of nine G6PD variants: G6PD Gaohe, G6PD Mahidol, G6PD Shoklo, G6PD Canton, G6PD Kaiping, G6PD Gaohe + Kaiping, G6PD Mahidol + Canton, G6PD Mahidol + Kaiping and G6PD Canton + Kaiping. All variants were less catalytically active and structurally stable than the wild type enzyme, with G6PD double mutations having a greater impact than single mutations. G6PD Shoklo and G6PD Canton + Kaiping were the least catalytically active single and double variants, respectively. The combined effects of two mutations were observed, with the Canton mutation reducing structural stability and the Kaiping mutation increasing it in the double mutations. Severe enzyme deficiency in the double mutants was mainly determined by the trade-off between protein stability and catalytic activity. Additionally, it was demonstrated that AG1, a G6PD activator, only marginally increased G6PD enzymatic activity and stability.

## Introduction

Glucose-6-phosphate dehydrogenase (G6PD) catalyzes the oxidation of glucose-6-phosphate (G6P) to 6-phosphogluconolactone with the formation of the reduced form of nicotinamide adenine dinucleotide phosphate (NADPH), a reducing power required to maintain oxidative balance in cells via reduced glutathione. G6PD is the sole producer of NADPH in red blood cells. As a result, G6PD deficiency can lead to a decrease in NADPH and reduced glutathione levels, causing oxidative damage^1^. Under this condition, red blood cells become susceptible to hemolysis. G6PD deficiency is an X-linked genetic disorder caused by a mutation(s) in the *G6PD* gene. G6PD deficiency is the most prevalent polymorphism and enzymopathy in humans, affecting approximately 500 million people worldwide^2^. Most identified G6PD variants are single-point mutations; however, the number of double and triple mutants reported is increasing^3–11^. Over 200 G6PD variants have been identified and are linked to a wide spectrum of clinical manifestations^12^. Some G6PD-deficient individuals are asymptomatic throughout their lifetime, whereas others experience clinical symptoms such as favism, hemolytic anemia, chronic non-spherocytic hemolytic anemia, spontaneous abortion, and neonatal hyperbilirubinemia resulting in neonatal kernicterus. Individuals with G6PD deficiency do not need treatment. However, they should avoid oxidative stressors in the form of infections, oxidative drugs, or fava beans^13^. Drug-induced hemolysis has raised serious concerns regarding malaria elimination because antimalarials, such as 8-aminoquinolines, have been linked to hemolytic anemia in G6PD-deficient individuals^14–17^. Furthermore, the prevalence of G6PD deficiency is high in regions where malaria is endemic, including Sub-Saharan Africa, the Arabian Peninsula and Asia which limits the use of 8-aminoquinolines^18,19^. Understanding the hemolytic risk of 8-aminoquinolines for G6PD-deficient patients is critical for safe and effective malaria treatment, which necessitates detailed knowledge of the patients G6PD mutation(s) and symptoms.

G6PD variants can be classified into 5 classes based on their residual enzymatic activity and clinical manifestations^20^. Among G6PD variants, different mutations cause varying degrees of enzyme deficiency, depending on the location of the mutations. Biochemical characterization has revealed that some G6PD variants affect catalytic efficiency, whereas others significantly affect protein stability^21–26^. Based on the three-dimensional (3D) structure, Class I G6PD variants such as G6PD Bangkok (Lys275Asn), G6PD Bangkok noi (Phe501Cys), G6PD Wisconsin (Arg393Gly) and G6PD Nashville (Arg393His), which cause severe enzyme deficiency, have amino acid substitutions clustered near the NADP^+^ binding site or the dimer interface, thereby disrupting the structural stability of G6PD^21,23,27,28^. Class II variants such as G6PD Viangchan (Val291Met) and G6PD Canton (Arg459Leu), associated with severe enzyme deficiency, are also found to affect protein stability^21,22^. Although the effect of a single mutation on protein function has been clearly demonstrated, the effects of multiple mutations on G6PD activity are not fully understood. In general, double and triple mutations cause more severe enzyme deficiencies than single mutations^7,29,30^. The level of enzyme activity in G6PD-deficient individuals carrying the double mutations G6PD Mahidol + Viangchan (Gly163Ser + Val291Met) was approximately tenfold lower than those carrying the individual mutations^7^. Furthermore, a combination of Asn126Asp (G6PD A) and Arg387His (G6PD Guadalajara) mutations causes the severe enzyme deficiency with hemolytic anemia observed in a Hispanic boy carrying G6PD Mount Sinai^29^. The detailed biochemical characterization of G6PD variants should provide important information on how the enzyme functions (efficiency of NADPH production), which reflects the response of the enzyme to oxidative stress. It is essential to understand how different mutations cause varying degrees of enzyme deficiency. Additionally, the information is useful for explaining the molecular mechanisms underlying the enzyme deficiency observed in G6PD-deficient individuals. Unfortunately, not all G6PD variants have been thoroughly characterized.

In this study, we investigated the effect of mutations on biochemical and structural properties of natural G6PD variants discovered in Southeast Asia ^8–11,31,32^. We examined five single variants [G6PD Gaohe (His32Arg), G6PD Mahidol (Gly163Ser), G6PD Shoklo (Ile234Thr), G6PD Canton (Arg459Leu) and G6PD Kaiping (Arg463His)], four double variants [G6PD Gaohe + Kaiping (His32Arg + Arg463His), G6PD Mahidol + Canton (Gly163Ser + Arg459Leu), G6PD Mahidol + Kaiping (Gly163Ser + Arg463His) and G6PD Canton + Kaiping (Arg459Leu + Arg463His)] and the wild type (WT) enzyme. We also assessed the interaction between two mutations in causing severe enzyme deficiency in the G6PD double variants. Recently, a small molecule activator of G6PD, an agonist of G6PD (AG1), was identified^33^. AG1 was shown to improve the activity of several common G6PD variants such as G6PD Canton, G6PD Mediterranean (Ser188Phe), G6PD A– (Val68Met + Asn126Asp) and G6PD Kaiping. Therefore, we also examined the effect of AG1 on enzyme activity and protein stability of the G6PD variants.

## Results

### Steady-state kinetic parameters of G6PD variants

The steady-state kinetic parameters were determined to assess the effect of mutations on the catalytic activity of G6PD variants (Table 1). All G6PD variants had a lower catalytic constant (*k*_cat_) when compared with that of the WT enzyme. Among single mutant variants, G6PD Mahidol (Gly163Ser) showed the smallest change in *k*_cat_ (approximately 30% reduction) when compared with the WT enzyme *k*_cat_, whereas G6PD Shoklo (Ile234Thr) was the most affected variant (almost 80% reduction). The *k*_cat_ values of Class II variants [G6PD Gaohe (His32Arg), G6PD Canton (Arg459Leu) and G6PD Kaiping (Arg463His)] were reduced by approximately 50% and those of the double mutations were lower than the single mutations with G6PD Gaohe + Kaiping (His32Arg + Arg463His) and G6PD Canton + Kaiping (Arg459Leu + Arg463His) having a 15-fold decrease when compared with the *k*_cat_ of G6PD WT. Although other variants showed similar binding affinity toward the G6P substrate when compared with the binding affinity of the WT enzyme, G6PD Canton, G6PD Mahidol + Canton (Gly163Ser + Arg459Leu) and G6PD Canton + Kaiping had lower *K*_m_ values (1.5–2.6-fold). The *K*_m_ for the NADP^+^ substrate was not significantly affected by any mutation. In terms of catalytic efficiency (*k*_cat_/*K*_m_), all variants were less catalytically active than the WT enzyme (two to tenfold), with double mutations having lower activity than single mutations. G6PD Shoklo was the least active single mutation (four to sixfold decrease), whereas G6PD Canton + Kaiping was the least active double mutation (~ tenfold decrease). The lower catalytic activity of the double mutants (Gaohe + Kaiping, G6PD Mahidol + Canton and G6PD Canton + Kaiping) despite higher binding affinity toward both substrates (G6P and NADP^+^) than the WT enzyme could be attributed to structural instability, which is discussed further below.

**Table 1:** Kinetic parameters of recombinant G6PD variants.

Construct	Class	Amino acid change	*k*_cat_ (s^–1^)	*K*_m_ G6P (µM)	*K*_m_ NADP^+^ (µM)	*k*_cat_/*K*_m_ G6P (µM^–1^ s^–1^)	*k*_*cat*_/*K*_m_ NADP^+^ (µM^–1^ s^–1^)
WT	–	–	296 ± 15	53 ± 7	10 ± 1	6 ± 1	29 ± 5
Gaohe	II	His32Arg	149 ± 11	48 ± 2	11 ± 3	3.9 ± 0.2	13 ± 4
Mahidol	III	Gly163Ser	214 ± 8	54 ± 3	14 ± 2	4.0 ± 0.3	15 ± 3
Shoklo	NR	Ile234Thr	69 ± 2	42 ± 7	9 ± 1	1.0 ± 0.2	7 ± 1
Canton	II	Arg459Leu	160 ± 3	20 ± 1	12 ± 3	8 ± 1	13 ± 3
Kaiping	II	Arg463His	147 ± 12	49 ± 8	16 ± 5	3 ± 1	9 ± 5
Gaohe + Kaiping	NR	His32Arg + Arg463His	22 ± 1	43 ± 4	7 ± 1	2.5 ± 0.3	3.2 ± 0.4
Mahidol + Canton	NR	Gly163Ser + Arg459Leu	79 ± 9	34 ± 3	12 ± 4	2.4 ± 0.3	7 ± 3
Mahidol + Kaiping	NR	Gly163Ser + Arg463His	101 ± 5	58 ± 7	16 ± 2	1.4 ± 0.2	6 ± 1
Canton + Kaiping	NR	Arg459Leu + Arg463His	20 ± 3	34 ± 3	6 ± 1	0.6 ± 0.1	3 ± 1

Experiments were performed in triplicate, and data are present as mean ± SD. *NR* no report.

### Effect of AG1 on enzyme activity of G6PD variants

**Figure 1:**
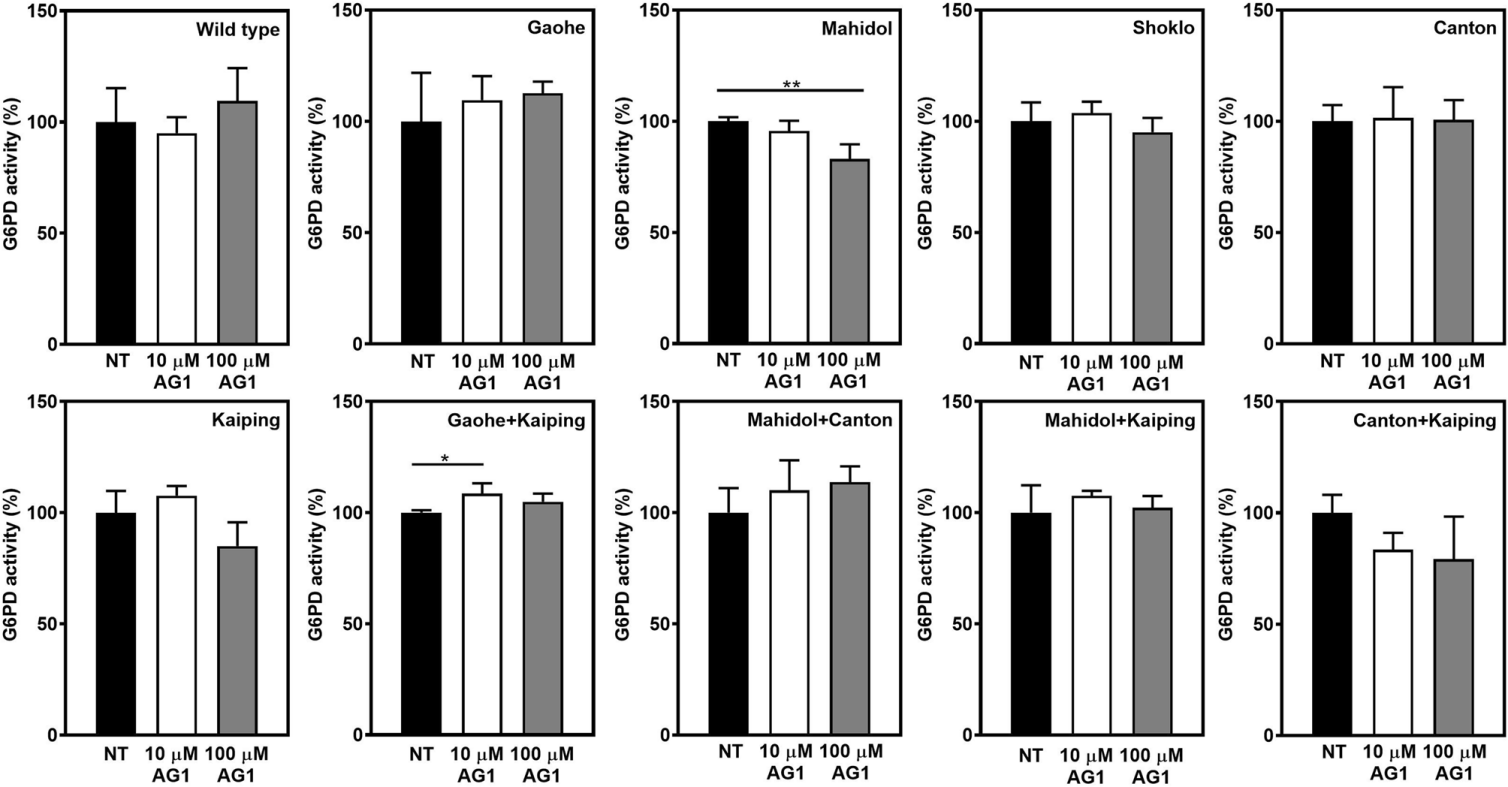
Effect of AG1 on the activity of G6PD variants. The enzyme was incubated with 10 and 100 μM of AG1 for 1 h at 4 °C. G6PD activity was measured and expressed as a percentage of the activity of the enzyme incubated in the absence of the AG1 activator or non-treated (NT). Statistical analysis was performed using one-way analysis of variance (ANOVA). Asterisks indicate statistical difference (*P ≤ 0.05 and **P ≤ 0.01) between groups. Error bars represent the standard deviation (SD) of the three experiments.

High-throughput screening has revealed that the compound AG1 increases the activity of G6PD WT and G6PD variants (G6PD Canton, G6PD Mediterranean, G6PD A^–^ and G6PD Kaiping)^33^. It was suggested that AG1 might promote the formation of active dimers or stabilize a dimeric form of G6PD. Herein, we examined the effect of AG1 (10 and 100 µM) on the activity of G6PD variants, including G6PD WT (Fig. 1 and Table S1). In the presence of 10 µM AG1, the activity of G6PD proteins was comparable to non-treated (NT, without AG1), except G6PD Gaohe + Kaiping which showed an increase in enzyme activity. Further increases the concentration of AG1 to 100 µM did not improve enzyme activity. In contrast, the higher concentration of AG1 (100 µM) caused a reduction in enzyme activity of G6PD Mahidol, G6PD Kaiping and G6PD Canton + Kaiping. Interestingly, in comparison to NT, AG1 (at both concentrations) inhibited the activity of G6PD Mahidol and G6PD Canton + Kaiping in a concentration-dependent manner. The decrease in enzymatic activity of these variants may indicate that AG1 has a destabilizing effect at higher concentrations (100 µM). The increase or decrease in enzymatic activity of G6PD variants was not statistically significant, except a decrease in activity of G6PD Mahidol at 100 µM and an increase in activity of G6PD Gaohe + Kaiping at 10 µM AG1.

### Secondary structure of G6PD variants by circular dichroism (CD)

**Figure 2:**
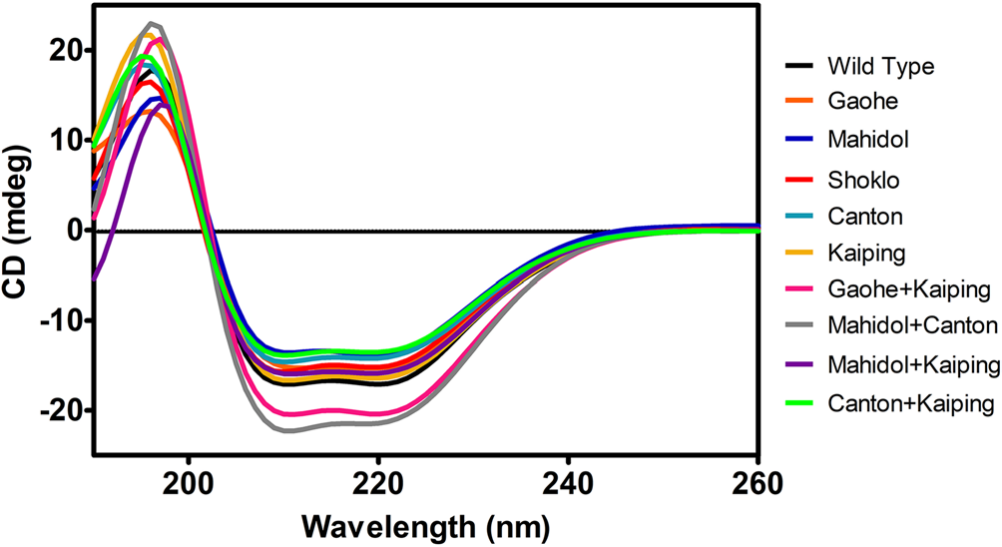
Far-UV spectra of recombinant G6PD variants. CD spectra were recorded over a wavelength range of 190–260 nm at a scan rate of 50 nm/min using a Jasco J-815 spectropolarimeter. Five scans were averaged and the buffer-only spectrum (20 mM Tris–HCl, pH 7.5) was subtracted.

Circular dichroism (CD) spectra in the far-ultraviolet (UV) region of the G6PD variants and WT were recorded to determine the effect of mutations on the secondary structure of G6PD variants (Fig. 2). The CD spectra for all G6PD variants were similar to the CD spectrum of the WT protein, with two-minima negative absorption peaks at 208 and 220 nm and a positive band at 193 nm. Such CD spectra are characteristic of α-helical proteins and in agreement with the structure of G6PD determined by X-ray crystallography^28^. All variants had lower signal intensities than the WT enzyme, except G6PD Gaohe + Kaiping and G6PD Mahidol + Canton which had stronger intensities, without changing the CD absorption pattern. Such intensity changes indicate a change in the chirality of the chromophores, which refers to the flexibility or rigidity of secondary structure elements. Although the G6PD Mahidol + Canton mutation did not affect the absorption pattern, the CD spectrum of this variant had a stronger negative minimum at 208 nm and thus changed the 222/208 nm ratio, which suggests an increase in the amount of random coil.

### Structural analysis of G6PD variants by intrinsic fluorescence and 8-anilinonaphthalene-1-sulfonate (ANS)-binding assays

**Figure 3:**
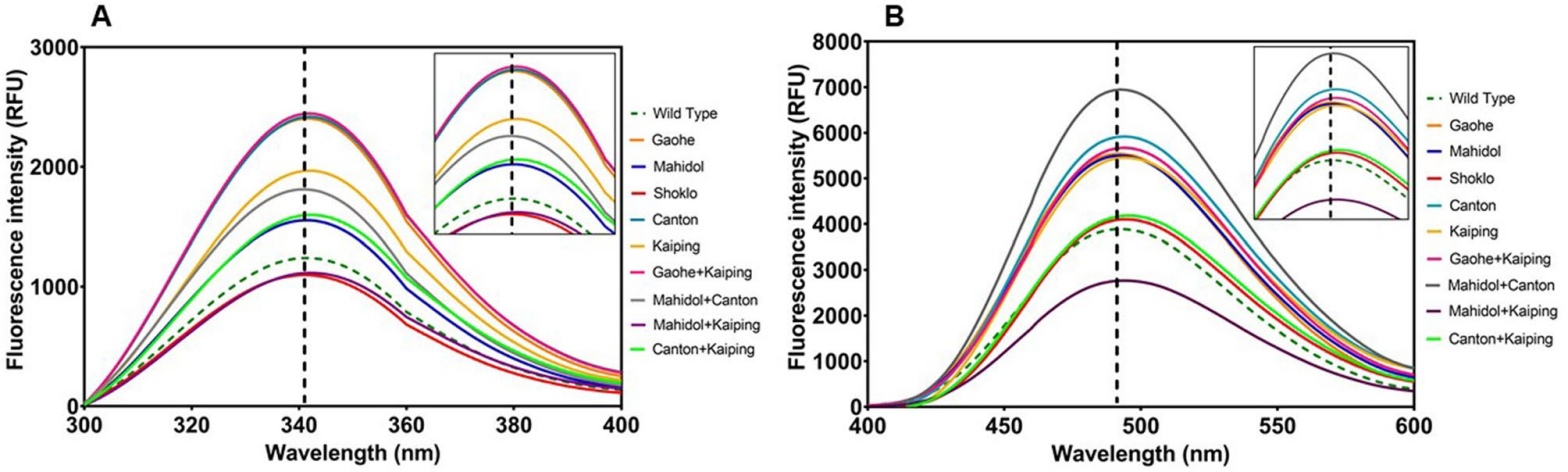
Fluorescence emission spectra of recombinant human G6PD variants. (**A**) Intrinsic fluorescence spectra. The emission spectra of G6PD proteins were recorded over the range of 300–400 nm using an excitation wavelength of 295 nm. (**B**) ANS fluorescence spectra. G6PD proteins were incubated with 100 μM ANS at 25 °C for 1 h and emission spectra were monitored between 400 and 600 nm using an excitation wavelength of 395 nm. The vertical dash-line represents the fluorescence emission maximum of the WT enzyme. Insets: magnified view of the fluorescence emission.

The presence of seven tryptophan residues in human G6PD can be used to probe changes in the 3D structure of G6PD variants. A change in the intrinsic fluorescence emission maxima provides useful information about changes in the microenvironment or molecular orientation of tryptophan residues. As shown in Fig. 3A, fluorescence emission spectra of G6PD Shoklo and G6PD Mahidol + Kaiping had weaker fluorescence intensities, whereas other G6PD variants exhibited stronger fluorescence intensities when compared with the spectrum of the WT enzyme. A slight shift of only 1 nm in the fluorescence emission maximum was observed for G6PD Gaohe, G6PD Canton, G6PD Kaiping, G6PD Gaohe + Kaiping, G6PD Mahidol + Kaiping and G6PD Canton + Kaiping when compared with the fluorescence emission maximum for G6PD WT. This small red shift in fluorescence emission maxima suggests that the hydrophobicity of the microenvironments for tryptophan residues in these variants has slightly decreased. Additionally, the emission fluorescence spectra of 8-anilinonaphthalene-1-sulfonate (ANS) were also recorded to determine the degree of structural perturbation caused by the mutations. The fluorescence signal of the amphiphilic dye ANS increases when exposed to a more hydrophobic environment. Only G6PD Mahidol + Kaiping exhibited a weaker ANS fluorescence signal when compared with that of G6PD WT. All other G6PD mutants gave a stronger ANS fluorescence signal than G6PD WT, with G6PD Mahidol + Canton giving the strongest ANS fluorescence signal (Fig. 3B). The ANS fluorescence emission maximum did not change for G6PD Gaohe and G6PD Mahidol mutants, whereas a small shift (1–3 nm) was observed for the other variants. A slight red shift suggests that these proteins are becoming more relaxed so that the ANS fluorophore has access to less polar environment.

### Structural stability of G6PD variants

#### Thermal stability of G6PD variants

**Figure 4:**
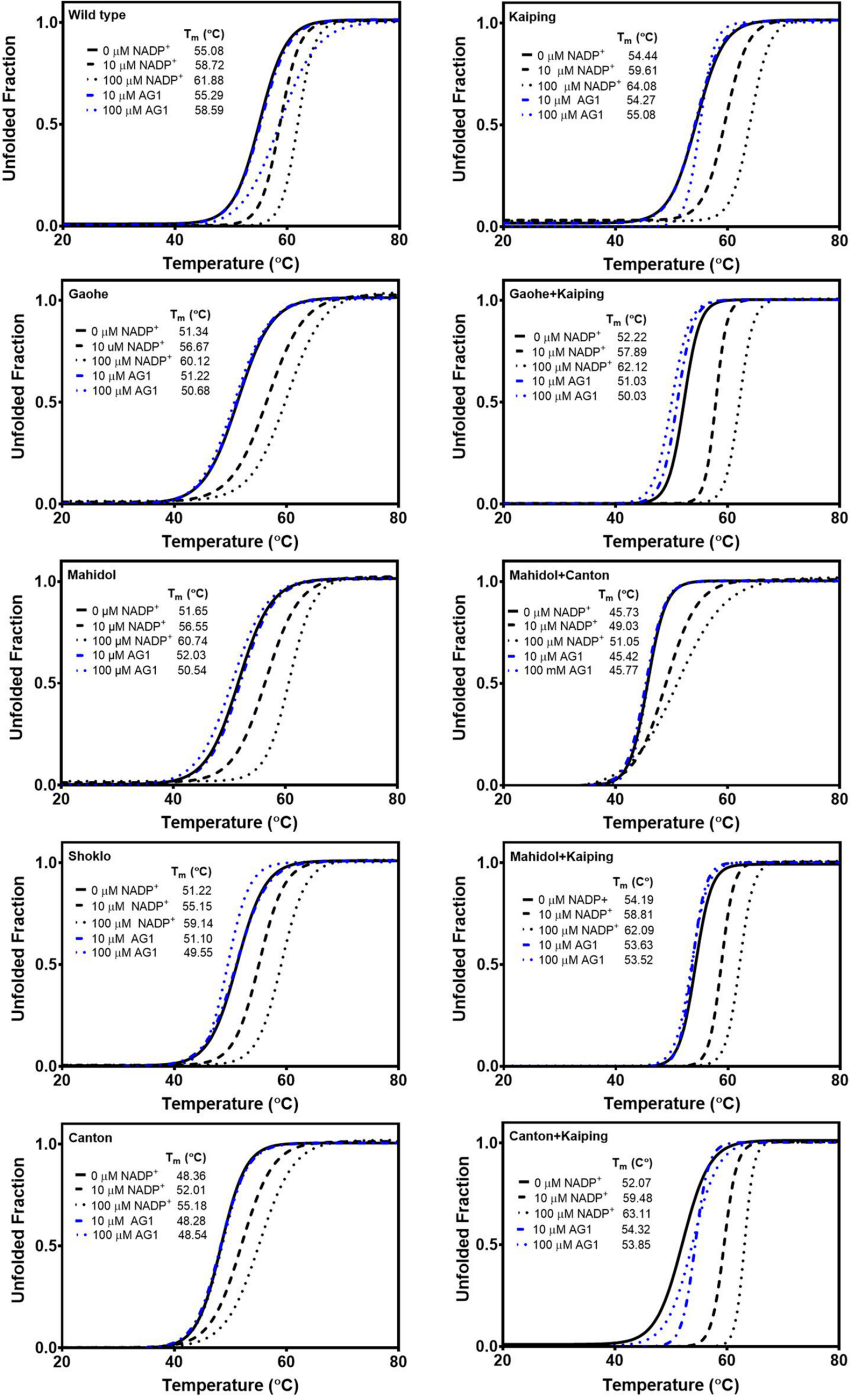
Thermal stability analysis of recombinant G6PD variants. G6PD proteins were heated (20 to 80 °C) in the presence of 5 × SYPRO Orange reporter dye and various concentrations of NADP^+^ (0, 10 and 100 μM) or AG1 (10 and 100 μM). Protein unfolding was monitored by measuring the emission of the fluorescence dye at 580 nm. Experiments were performed in triplicate, and the data were averaged and plotted before calculating the *T*_m_ using GraphPad Prism.

Fluorescence-based thermal shift assays were carried out in the absence and presence of different concentrations of NADP^+^ (0, 10 and 100 μM) or AG1 (0, 10 and 100 μM) to assess the structural stability of G6PD variants. Upon increasing the temperature, the protein structure unfolded and the temperature at which half of the protein was unfolded was defined as *T*_*m*_. As shown in Fig. 4 and Table S2, in the absence of NADP^+^, G6PD WT was the most stable protein with a *T*_m_ value of 55.1 °C, whereas the G6PD variants were less stable with G6PD Mahidol + Canton being the least stable variant (*T*_m_ = 45.7 °C). In agreement with previous reports^21–23^, the presence of NADP^+^ enhanced the structural stability of all G6PD proteins significantly in a concentration-dependent manner. Noteworthy, in the absence of NADP^+^, the *T*_m_ value of G6PD Kaiping (54.4 °C) was only 0.6 °C lower than the *T*_m_ for G6PD WT, and the presence of NADP^+^ (10 and 100 μM) markedly increased protein stability, yielding *T*_m_ values even greater than the WT enzyme. At 100 μM NADP^+^, the *T*_m_ values were 61.9 °C and 64.1 °C for G6PD WT and G6PD Kaiping, respectively. Additionally, the presence of the Kaiping mutation also contributed to the greater structural stability of the double mutation variants when compared with the corresponding single mutation variants.

On the contrary, the *T*_m_ values of all G6PD proteins in the presence of 10 μM AG1 were similar to the *T*_m_ values in the absence of AG1, with the exception of a decrease (1.2 °C) and increase (2.3 °C) in *T*_*m*_ values for G6PD Gaohe + Kaiping and G6PD Canton + Kaiping, respectively. In the absence of AG1, the *T*_*m*_ values were 52.2 °C and 52.1 °C for G6PD Gaohe + Kaiping and G6PD Canton + Kaiping respectively. AG1 present at 100 μM did not affect the structural stability of G6PD Canton (*T*_m_ = 48.5 °C) and G6PD Mahidol + Canton (*T*_m_ = 45.8 °C), whereas a small effect was observed for G6PD Kaiping (*T*_m_ = 55.1 °C) and G6PD Canton + Kaiping (*T*_m_ = 53.9 °C), with an increase in *T*_m_ of 0.6 °C and 1.8 °C, respectively. Interestingly, 100 μM AG1 improved the structural stability of G6PD WT with the *T*_m_ increasing to 58.6 °C. In contrast, this small molecule destabilized the structure of G6PD Gaohe, G6PD Mahidol, G6PD Shoklo, G6PD Gaohe + Kaiping and G6PD Mahidol + Kaiping slightly, with *T*_m_ values decreasing by 0.7‒2.2 °C when compared with the *T*_m_ values determined in the absence of this activator.

#### Thermal inactivation of G6PD variants

**Figure 5:**
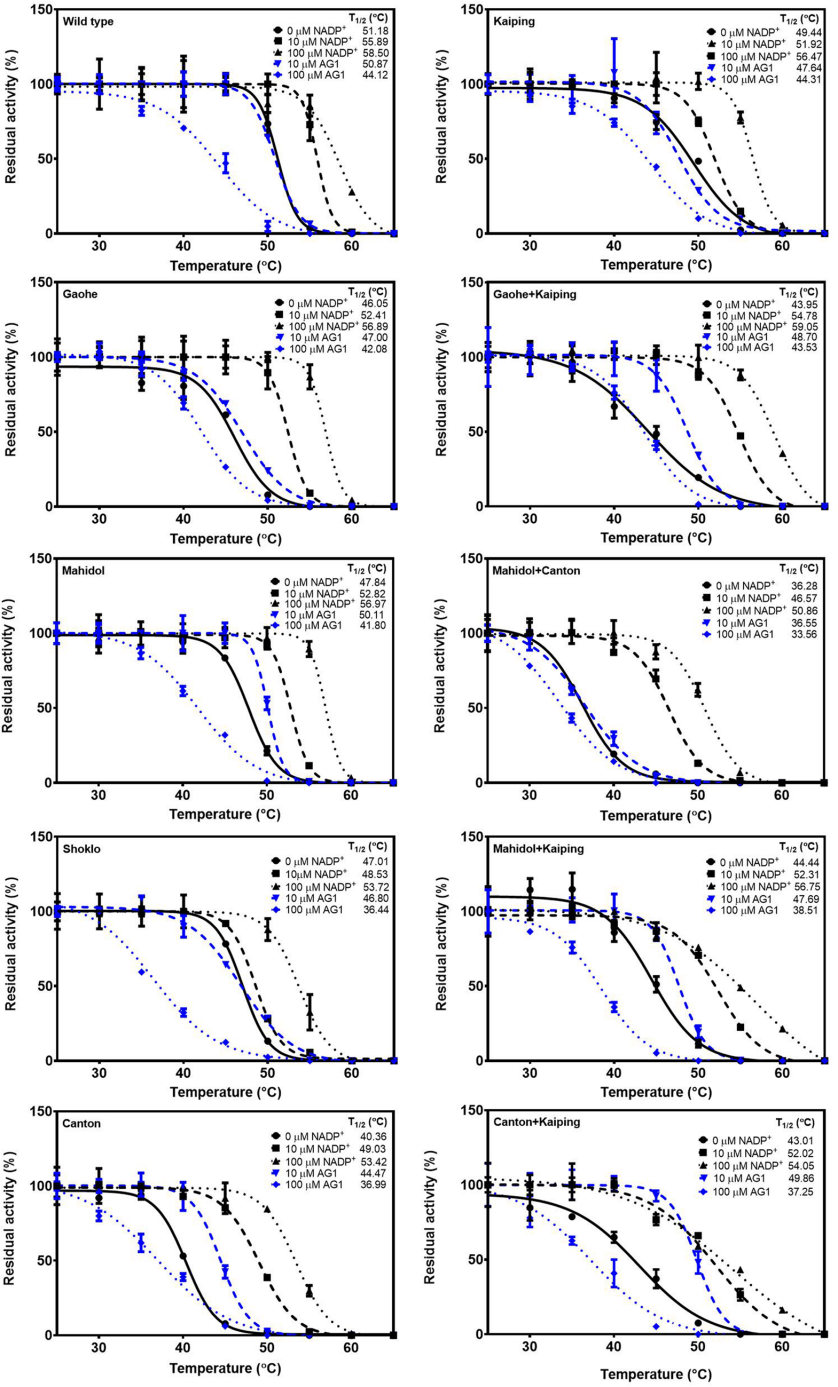
Thermal inactivation analysis of recombinant G6PD variants. Residual enzyme activity was measured after the protein was heated at different temperatures (25 to 65 °C) for 20 min in the presence of various concentrations of NADP^+^ (0, 10 and 100 μM) or AG1 (10 and 100 μM). *T*_1/2_ is the temperature at which the enzyme loses 50% activity. Error bars represent mean ± SD of triplicate measurements.

Thermal inactivation assays were carried out to evaluate the effect of mutations on the stability of G6PD variants upon heat treatment (Fig. 5 and Table S3). Residual enzyme activity was measured after 20-min exposure to temperatures in the range of 25‒65 °C, and expressed as a percentage of the activity of the same enzyme incubated at 25 °C. *T*_1/2_ was defined as the temperature at which the enzyme lost 50% of its activity. In agreement with the thermal stability assays, G6PD WT was the most stable protein in the absence of a ligand, and G6PD Mahidol + Canton displayed the lowest thermotolerance with *T*_1/2_ values of 51.2 and 36.3 °C, respectively. The presence of NADP^+^ was found to improve the structural stability of the protein, resulting in an increase in the *T*_1/2_ values for all G6PD variants and the WT protein. The stabilizing effect of NADP^+^ was concentration-dependent and clearly noticeable for G6PD Canton, G6PD Gaohe + Kaiping, G6PD Mahidol + Canton and G6PD Canton + Kaiping, with *T*_1/2_ increasing by more than 8 °C when compared with the results recorded in the absence of NADP^+^. In comparison to single mutants, a greater stabilizing effect of NADP^+^ was observed for the double mutants. The presence of 10 μM AG1 increased *T*_1/2_ values of all variants, except G6PD WT, G6PD Shoklo and G6PD Kaiping. In contrast, the higher AG1 concentration (100 μM) destabilized the protein structure, resulting in lower *T*_1/2_ values of all proteins, with G6PD Shoklo being the most affected variant (10.6 °C reduction). In contrast to thermal stability analysis, the presence of the Kaiping mutation did not contribute to structural stability of the proteins upon heat treatment.

#### Stability of G6PD variants upon treatment with guanidine hydrochloride (Gdn-HCl)

**Figure 6:**
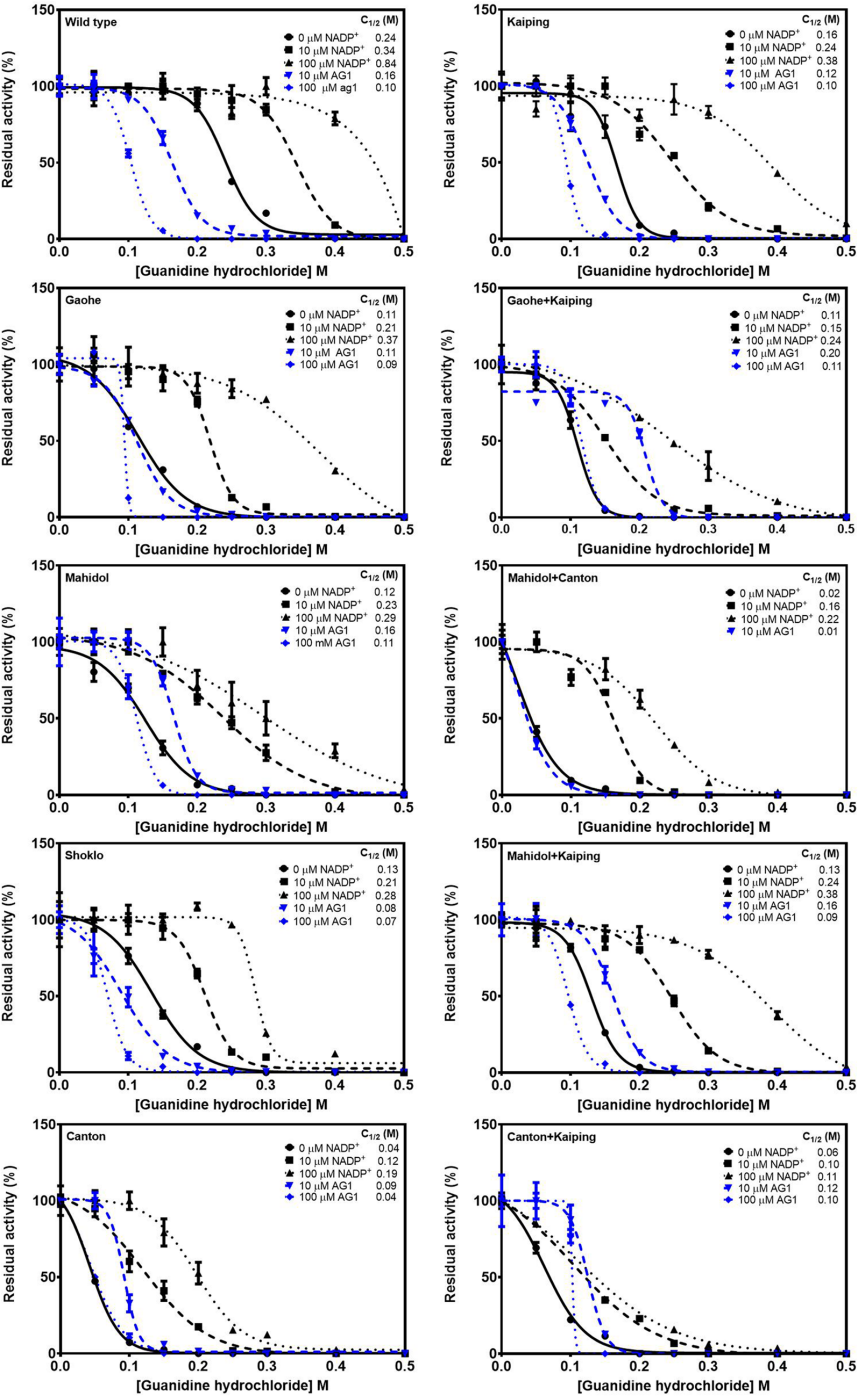
Stability analysis of recombinant G6PD variants upon Gdn-HCl treatment. Residual enzymatic activity was determined after incubation with different concentrations of Gdn-HCl (0‒0.5 M) at 37 °C for 2 h in the presence of various concentrations of NADP^+^ (0, 10 and 100 μM) or AG1 (10 and 100 μM). Residual enzyme activity is expressed as a percentage of the activity for the same enzyme incubated in the absence of Gdn-HCl. *C*_1/2_ is the Gdn-HCl concentration at which the enzyme loses 50% of its activity. Error bars represent mean ± SD of triplicate measurements.

Protein unfolding during exposure to a chemical denaturant can be used to investigate the structural stability of proteins. Thus, the stability of G6PD variants was assessed upon treatment with increasing concentrations of guanidine hydrochloride (Gdn-HCl; 0 to 0.5 M). Residual enzyme activity was measured and expressed as a percentage of the activity for the same enzyme incubated without Gdn-HCl. *C*_1/2_ was defined as the Gdn-HCl concentration at which the enzyme loses 50% of its activity, and the results are shown in Fig. 6 and Table S4. In agreement with thermal stability and heat inactivation analyses, in the absence of NADP^+^, G6PD WT was the most structurally stable protein upon treatment with Gdn-HCl with a *C*_1/2_ of 0.24 M. G6PD Canton was the least tolerant variant and G6PD Kaiping was the most tolerant variant to Gdn-HCl among the single G6PD variants examined with *C*_1/2_ values of 0.04 and 0.16 M, respectively. As expected, for double mutation variants, G6PD Mahidol + Canton was the most susceptible to Gdn-HCl denaturation with a *C*_1/2_ of 0.02 M. The presence of the Kaiping mutation did not improve the structural stability of the double mutants to Gdn-HCl treatment. The stabilizing effect of NADP^+^ was observed in a concentration-dependent fashion for all proteins. The presence of 10 μM AG1 stabilized G6PD Mahidol, G6PD Canton, G6PD Gaohe + Kaiping, G6PD Mahidol + Kaiping and G6PD Canton + Kaiping upon Gdn-HCl treatment, whereas the compound destabilized G6PD WT, G6PD Shoklo, G6PD Kaiping and G6PD Mahidol + Canton when compared with data in the absence of AG1 or NADP^+^. Notably, 100 μM AG1 largely destabilized G6PD Mahidol + Canton, in which the enzyme activity was too low to detect, and determination of *C*_1/2_ was not possible.

#### Susceptibility of G6PD variants to trypsin digestion

**Figure 7:**
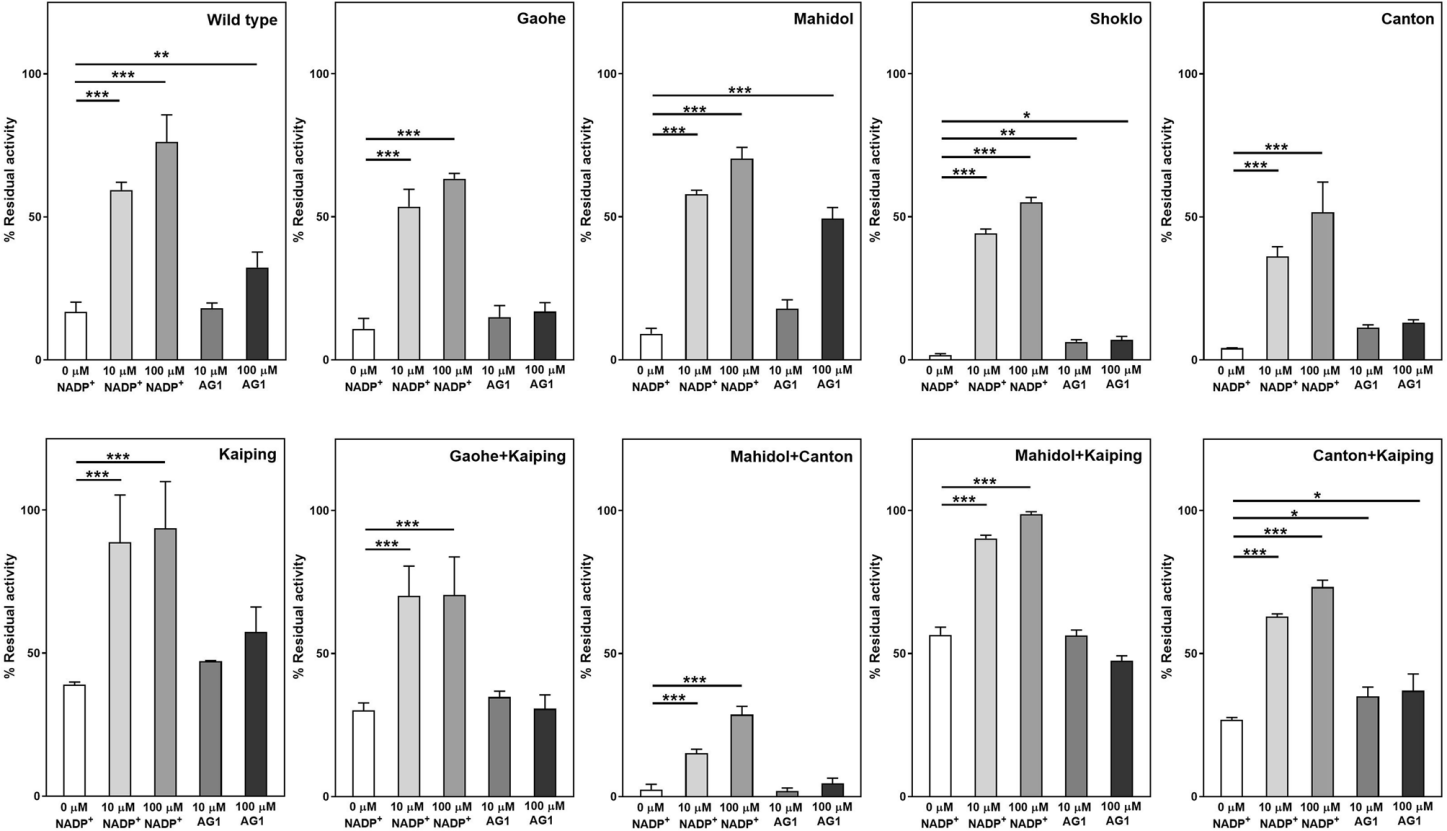
Susceptibility of recombinant G6PD variants to trypsin digestion. Residual enzymatic activity was measured after incubation with 0.5 mg/ml trypsin at 25 °C for 5 min in the presence of various concentrations of NADP^+^ (0, 10 and 100 μM) or AG1 (0, 10 and 100 μM). Residual enzyme activity was expressed as a percentage of the activity for the same enzyme in the absence of trypsin. Statistical analysis was performed using one-way analysis of variance (ANOVA). Asterisks indicate statistical difference (*P ≤ 0.05, **P ≤ 0.01 and ***P ≤ 0.001) between groups. Error bars represent the mean ± SD of triplicate measurements.

To further evaluate the structural stability of G6PD variants, proteolytic susceptibility assays were performed with trypsin in the absence and presence of different concentrations of NADP^+^ (0, 10 and 100 μM) or AG1 (10 and 100 μM). Residual enzymatic activity was measured after incubation with 0.5 mg/mL trypsin and expressed as a percentage of the activity for the same enzyme in the absence of trypsin (Fig. 7 and Table S5). Among single G6PD variants in the absence of NADP^+^, only the G6PD Kaiping retained greater activity (39%) when compared with that of G6PD WT (17%). For the double mutants, as expected, G6PD Mahidol + Canton showed greater susceptibility to trypsin digestion (residual activity 3%), whereas those containing the Kaiping mutation showed greater resistance to trypsin digestion than the WT enzyme, with residual activity of 27‒57%. The presence of AG1 and NADP^+^ provided a protective effect against trypsin digest in concentration-dependent manner for G6PD enzymes, and this protective effect was strongest when NADP^+^ was present. However, it should be noted that G6PD Gaohe + Kaiping and G6PD Mahidol + Kaiping were slightly more susceptible to trypsin digestion in the presence of AG1.

### Oligomeric state of G6PD variants

**Figure 8:**
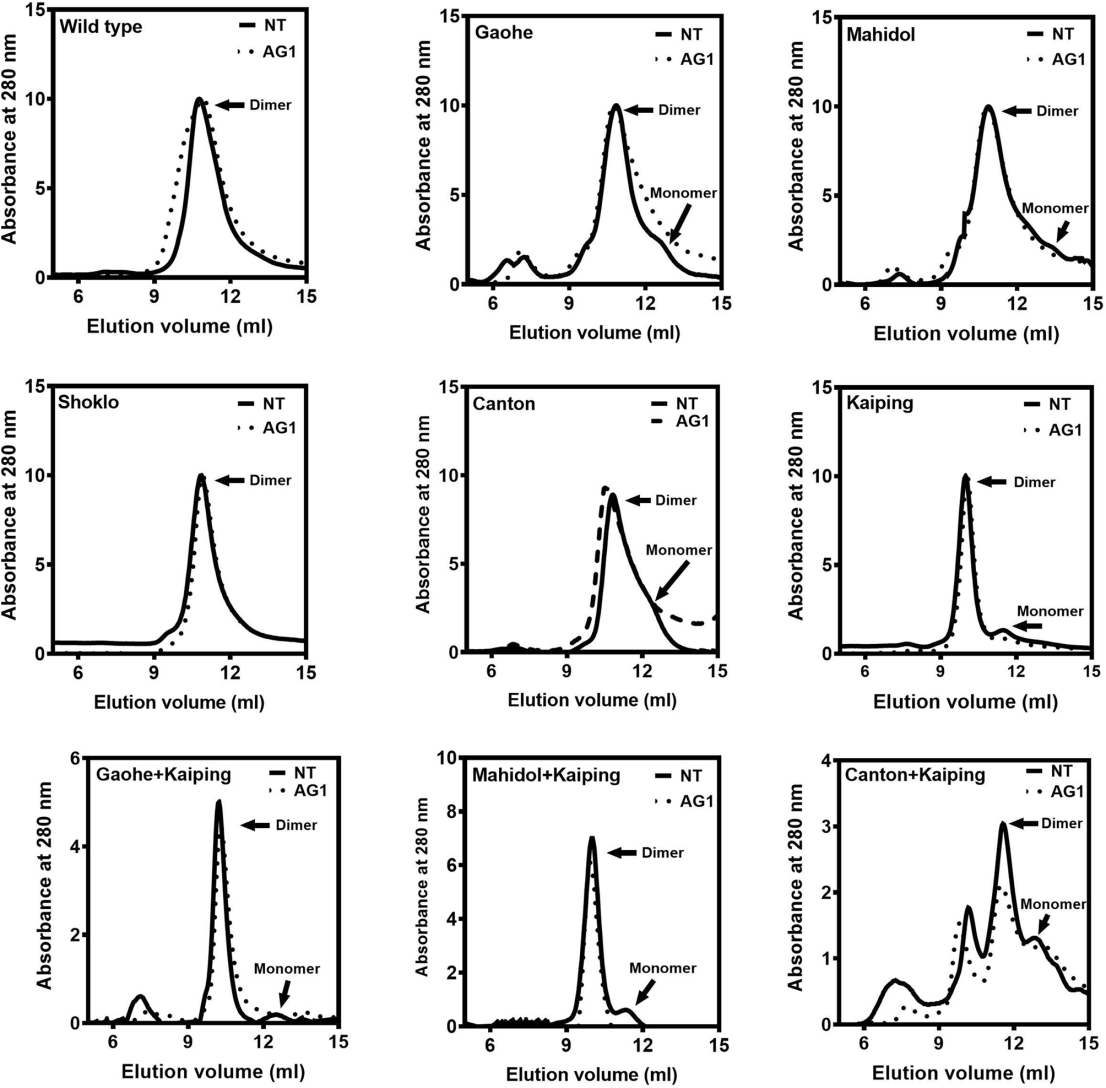
Size exclusion FPLC elution profiles of purified recombinant G6PD variants in the presence and absence of 500 μM AG1. Proteins were loaded onto Superdex 200 Increase 10/300 equilibrated with 50 mM Tris–HCl and 150 mM NaCl.

Native human G6PD is active as a dimer or tetramer^28^. Thus, size exclusion chromatography was carried out to determine whether the mutations affected the oligomeric state of G6PD variants in the absence and presence of 500 μM AG1. As shown in Fig. 8, in the absence of AG1, G6PD WT and G6PD Shoklo eluted as a single peak, corresponding to a dimeric form. In contrast, other G6PD variants eluted predominantly as dimers with a peak or shoulder of weaker intensity that represented an inactive monomer. The presence of 500 μM AG1 promoted dimerization of G6PD Gaohe, G6PD Kaiping, G6PD Gaohe + Kaiping and G6PD Mahidol + Kaiping. Unfortunately, G6PD Mahidol + Canton was structurally unstable, and size exclusion chromatogram data were not obtained.

## Discussion

G6PD deficiency is caused by a mutation(s) in the *G6PD* gene with over 200 variants reported worldwide, and each mutation has a different effect that results in a wide range of clinical manifestations^12,34^. Two major mechanisms contributing to enzyme deficiency observed in G6PD-deficient individuals are decreased catalytic efficiency and structural instability, depending on the location of the mutation in 3D structure of G6PD. Herein, the molecular mechanisms of G6PD enzyme deficiency of nine G6PD variants was systematically and extensively investigated, and the WT enzyme was included as a control.

Among single mutants, while a small effect was observed for other variants, the Shoklo mutation (Ile234Thr) was found to drastically affect the catalytic efficiency of G6PD, resulting in a fourfold reduction in catalytic activity without altering the binding affinity toward the two substrates. Based on the 3D structure, the Ile234Thr mutation is located in the structural NADP^+^ binding site (Fig. S1A). The structural NADP^+^ has been known to maintain G6PD catalytic activity by stabilizing the dimeric state. The pathogenic mutations located around the structural NADP^+^ binding site were found to be structurally less stable than the G6PD WT^35,36^. These mutations result in the loss of structural NADP^+^ and the disorder of the C-terminal tail, which blocks the G6P-binding site through long-range commination and reduces the enzyme activity. CD analysis revealed that the Ile234Thr mutation did not alter the secondary structure of G6PD, although a slight effect on the intrinsic and extrinsic fluorescence signal of the protein was observed. Furthermore, reduced catalytic activity was not caused by dissociation of the catalytically active form (dimer), as shown by size exclusion chromatography. In particular, the Shoklo mutation was found to reduce protein stability because a reduction in *T*_m_ and greater susceptibility to Gdn-HCl and trypsin treatment when compared with those of the WT enzyme were observed. G6PD Shoklo was identified in a heterozygous woman among Karen and Burman living in Thailand^37^. This variant has not been classified by the World Health Organization and has not been characterized previously. This study was the first to report detailed functional and structural characteristics of G6PD Shoklo, in which the Ile to Thr amino acid mutation reduced protein stability and caused a decrease in catalytic efficiency. Furthermore, based on the results, G6PD Shoklo displayed similar structural characteristics to G6PD Gaohe and G6PD Mahidol, which belong to Class II and Class III variants, respectively. However, G6PD Shoklo was less catalytically active than G6PD Gaohe and G6PD Mahidol. Therefore, G6PD Shoklo should probably be classified as a Class II variant. However, more clinical data such as residual enzyme activity in red blood cells and clinical phenotypes from individuals carrying the Ile234Thr mutation are required to confirm the preliminary classification suggested.

G6PD Gaohe (His32Arg), a Class II variant, has been reported in Chinese and Asian populations^38–41^. A previous report on functional characterization of G6PD Gaohe revealed that the mutation caused a 90% decrease in enzyme activity with an approximately threefold reduction in *K*_m_ values for both substrates^42^. In contrast, we observed that the His32Arg mutation had no effect on *K*_m_ for either substrate but reduced the catalytic efficiency by two-fold. The discrepancies between the two studies may arise from the different experimental conditions used. Previously, the enzyme activity was measured at pH 7.4 with much higher concentrations of NADP^+^ (0.20–2.4 mM) and G6P (0.20–2.0 mM)^42^. For structural characterization, our results showed that G6PD Gaohe was structurally less stable than the WT enzyme. Based on intrinsic tryptophan fluorescence and extrinsic tryptophan fluorescence in the ANS-binding assay, the His32Arg mutation perturbed the 3D structure of the protein. A small peak corresponding to an inactive monomeric form was also observed by size exclusion chromatography analysis, suggesting that structural instability of the G6PD Gaohe causes partial dissociation of the active dimer. The effect of the His32Arg mutation on thermal stability was moderate and similar to that observed for G6PD Mahidol and G6PD Shoklo.

For other single mutants, G6PD Mahidol (Gly163Ser), G6PD Canton (Arg459Leu) and G6PD Kaiping (Arg463His) were less catalytically active than the WT enzyme. The Canton mutation caused a 2.5-fold decrease in *K*_m_ for G6P, whereas the other two variants did not significantly alter the binding affinity toward both substrates when compared with that of G6PD WT. The three mutations have different effects on the structural stability of G6PD. Changes in the fluorescence intensities of tryptophan and ANS indicated that these three mutations do not disturb the secondary structure but alter the 3D structure of the protein. The Mahidol mutation, a Class III variant, caused moderate structural instability. In contrast, the Canton mutation, a Class II variant, induced significant structural instability, resulting in markedly lower thermostability and remarkably lower tolerance to Gdn-HCl and trypsin digestion when compared with that of the WT protein. The Canton mutation was found to cause loosening of inter-helical interactions, resulting in changes in the orientation of key residues (Lys171 and Pro172) involved in positioning of G6P and NADP^+^ in their binding pockets^33^. This finally leads to decreased enzyme activity and stability of this variant. Thermal inactivation and susceptibility to Gdn-HCl indicated that the Kaiping mutation (a Class II variant) affected the structural stability of the protein moderately. Interestingly, in the absence of NADP^+^, G6PD Kaiping had a similar *T*_m_ to that of G6PD WT; however, when NADP^+^ was present, the *T*_m_ value of G6PD Kaiping was higher than that of G6PD WT, indicating a greater stabilizing effect of NADP^+^ for this mutant when compared with that of the WT enzyme. Moreover, upon trypsin digestion, G6PD Kaiping retained higher residual enzyme activity than G6PD WT under all conditions tested, indicating greater resistance to proteolytic digestion. This resistance may be due to removing a trypsin cleavage site when Arg was substituted with His at residue 463. Conversely, the substitution of Arg with Leu at residue 459 of G6PD Canton did not afford greater resistance to trypsin digestion despite the Arg459Leu mutation removing a potential trypsin cleavage site. Inspection of the structure reveals that the Arg459Leu mutation is buried and inaccessible to trypsin (Fig. S1B). The observed severe enzyme deficiency of Class II variants (G6PD Canton and G6PD Kaiping) may also arise from the dissociation of active dimers into an inactive monomer species.

A greater effect on catalytic efficiency and structural stability was observed when two mutations were present in G6PD variants. The double mutant variants were less catalytically active than G6PD WT and single mutants. Because of the Canton mutation, a small decrease in *K*_m_ for G6P was observed for the G6PD Mahidol + Canton (Gly163Ser + Arg459Leu) and G6PD Canton + Kaiping (Arg459Leu + Arg463His) variants. The structural stability of the G6PD Mahidol + Canton variant was most affected when compared with G6PD WT. In terms of secondary structure, G6PD Gaohe + Kaiping (His32Arg + Arg463His) and G6PD Mahidol + Canton had a greater CD intensity than other proteins. In fact, a change in signal intensity without a change in the CD spectral pattern indicates modifications to the chirality of the chromophores caused by the mutation, which reflects on the stability of the secondary structure^23^. Interestingly, the CD spectrum of G6PD Mahidol + Canton showed a change in the 222/208 nm ellipticity, which indicated an increase in the amount of random coil^43^. This is consistent with structural analysis, which found that the Canton mutation caused loosening of inter-helical interactions and helix displacements^33^.

In accordance with that observed for individual mutations, the double mutants containing the Kaiping mutation (G6PD Gaohe + Kaiping, G6PD Mahidol + Kaiping (Gly163Ser + Arg463His) and G6PD Canton + Kaiping) showed greater thermostability than the double mutant without the Kaiping mutation (G6PD Mahidol + Canton). The presence of the Kaiping mutation also increased the resistance to trypsin digestion of the double mutants, and these mutants were more resistant to trypsin than that of G6PD WT. When enzyme stability was assessed using different approaches (e.g. thermostability, susceptibility to Gdn-HCl and trypsin digestion), all the variants gave consistent results, with G6PD Mahidol + Kaiping having greater structural stability than G6PD Gaohe + Kaiping, G6PD Canton + Kaiping and G6PD Mahidol + Canton, respectively. According to size exclusion chromatography, all double mutants existed in monomeric and dimeric forms, suggesting these mutations reduce the stability of the dimer. This shift to a partial population of inactive monomers caused a decrease in enzyme activity for the double variants when compared with that of the WT enzyme. Notably, the combined Canton and Kaiping mutations caused the largest reduction in dimer stability.

The loss of enzyme function of G6PD variants can occur via different biochemical mechanisms, resulting in a wide range of clinical manifestations. It was shown here that the overall fitness of G6PD variants (e.g. good catalytic efficiency and structural stability) attributed to enzyme deficiency. The catalytic activity or structural stability alone can not be used to determine or indicate the clinical phenotypes. Indeed, a combination of both factors contributes to the severity of G6PD deficiency and, as a result, is an important determinant for classifying G6PD variants. A trade-off between stability and activity were observed for all G6PD variants studied here. Similarly, a coupling between stability and activity has been reported for other G6PD variants^21,44^.

NADP^+^ binds human G6PD and plays an essential role in stabilizing the protein^28,45,46^. In good agreement with previous studies, NADP^+^ was demonstrated to stabilize all G6PD variants^21–23,25,26^. The presence of 10 and 100 μM NADP^+^ increased thermostability and resistance to trypsin digestion and Gdn-HCl treatment significantly and in a concentration-dependent manner.

AG1 was identified previously through a high-throughput screening assay using recombinant G6PD Canton as a mild small molecule activator of G6PD^33^. AG1 was reported to increase the activity of G6PD WT by ~ 20% and G6PD Canton by 1.7-fold with an EC_50_ of approximately 3 μM. AG1 also increased the enzyme activity (up to twofold) of other variants (G6PD Mediterranean, G6PD A^–^ and G6PD Kaiping). However, it was shown here that the effect of AG1 on G6PD activity was only marginal and insignificant, 2‒10% and 1‒14% at 10 and 100 μM, respectively. The highest concentration of AG1 did not always improve G6PD activity of the variants with the activity of G6PD Mahidol, G6PD Shoklo, G6PD Kaiping, and G6PD Canton + Kaiping reduced in the presence 100 μM AG1. In particular, AG1 reduced the enzyme activity of G6PD Mahidol and G6PD Canton + Kaiping in a concentration-dependent manner. The effect of AG1 on promoting G6PD enzyme activity was found to be very small and insignificant, which differs from a previous report^33^. This discrepancy may be because a different assay was used to determine G6PD enzyme activity; a direct measure of NADPH production was carried out in this study, whereas a coupled assay with diaphorase to generate fluorescent resorufin was used previously. Additionally, the standard reaction conditions differed between the two studies; much lower concentrations of NADP^+^ (10 μM) and G6P (100 μM) were used previously. The kinetic parameters from the previous study of G6PD WT and G6PD Canton were also quite different when compared with those from other studies^21,27,33^. Nevertheless, it was suggested that AG1 increased G6PD enzyme activity by promoting/stabilizing active dimer levels^33,47^. In agreement with this observation, the presence of AG1 was also found to enhance the formation of the active dimer of G6PD Gaohe, G6PD Kaiping, G6PD Gaohe + Kaiping, and G6PD Mahidol + Kaiping.

In terms of structural stabilization, AG1 enhanced the thermostability of G6PD variants slightly. AG1 present at 10 μM increased *T*_m_ values of G6PD Mahidol and G6PD Canton + Kaiping by 0.4 and 2.3 °C, respectively and 100 μM AG1 increased *T*_m_ values by 3.5 °C for G6PD WT and 0.6 °C for and G6PD Kaiping. In contrast, 10 μM AG1 increased *T*_1/2_ values (0.3‒6.9 °C) of all variants with the exception of G6PD WT, G6PD Shoklo and G6PD Kaiping, whereas 100 μM AG1 decreased *T*_1/2_ values of all G6PD variants. Furthermore, AG1 mildly increased resistance of the G6PD variants to Gdn-HCl denaturation and trypsin digestion. The results indicate that AG1 mainly improved the enzyme activity of G6PD variants by promoting the formation of the active dimer, whereas AG1 did not improve protein stability significantly.

## Conclusions

In this study, biochemical and structural characteristics of nine G6PD variants (five single and four double mutations) were examined. All G6PD variants were less catalytically active than the WT enzyme, with double mutations having a greater impact on catalytic efficiency than single mutations. Alterations in the catalytic activity of the double mutants may be attributed to the presence of both mutations. The mutations studied here had varying effects on the structural stability of G6PD variants. Structural instability was increased noticeably by the G6PD Canton mutation, whereas G6PD Kaiping afforded greater resistance to trypsin digestion when compared with that of the WT enzyme. The Kaiping mutation also contributed to greater structural stability of the double mutation variants when compared with the corresponding single mutation variants. It was observed that severe enzyme deficiency in the double mutants was mainly determined by the trade-off between protein stability and catalytic activity. Although NADP^+^ significantly improved the structural stability of G6PD variants, AG1, a small molecule activator of G6PD, only marginally improved the stability of the G6PD variants. AG1 enhanced enzyme activity by promoting/stabilizing the dimeric form of the G6PD variants. Based on the presented biochemical and structural results, we recommend that G6PD Shoklo and all double mutations be classified as Class II variants because G6PD Shoklo was less catalytically active than other Class II variants studied herein and double mutations caused significant structural instability.

## Methods

### Construction of recombinant G6PD variants by site-directed mutagenesis

Recombinant plasmid, pET28a-G6PD WT, was used as a template to create the desired mutations by site-directed mutagenesis. The polymerase chain reaction (PCR) mixture (50 μL) contained 1 × reaction buffer, 50 ng of template plasmid, 100 ng of forward and reverse primers, 0.3 μM of each dNTP, 1 U of Phusion High-Fidelity DNA polymerase (New England Biolabs, Inc., Beverly, MA, USA.) and 3% DMSO. The primers used for site-directed mutagenesis are listed in Table S6. The cycling parameters are as follows: 1 cycle of 98 °C for 3 min and 25 cycles of 98 °C for 30 s, 45 °C for 1 min, and 68 °C for 3 min and 30 s. PCR products were digested with *Dpn*I to remove the parental plasmid and were then transformed into DH5α competent cells. The presence of the desired mutations was verified by DNA sequencing. For protein expression, recombinant plasmids with desired mutations were transformed into the BL21 (DE3) expression host.

### Protein expression and purification of G6PD variants

Expression and purification of G6PD variants were performed as previously described ^21–23^. Fresh overnight cultures of *E. coli* BL21 (DE3) harboring recombinant plasmid were grown at 37 °C with 250 rpm shaking in LB media containing 50 μg/ml kanamycin. G6PD expression was induced with isopropyl β-D-thiogalactopyranoside at a final concentration of 1 mM when the OD_600_ reached 1.0. The cultures were then incubated at 20 °C for 20 h with 200 rpm shaking before being harvested by centrifugation at 8300 × g for 10 min.

For protein purification, the pellets were resuspended in lysis buffer (20 mM sodium phosphate buffer pH 7.4, 10 mM imidazole, and 300 mM NaCl), disrupted by sonication for 5 min, and centrifuged at 36,000 × g for 1 h. Protein was purified using immobilized metal affinity chromatography by incubating with TALON cobalt resin (Takara Bio, Shiga, Japan) for 1 h. The unbound proteins were removed using wash buffer (20 mM sodium phosphate buffer pH 7.4, 20 mM imidazole, and 300 mM NaCl). Then, the protein was eluted using an imidazole concentration gradient (40–400 mM) and the fractions containing the desired protein were pooled. Overnight dialysis against 20 mM Tris–HCl pH 7.5 containing 10% glycerol was carried out to remove imidazole. The purity of the obtained protein was determined by 12% SDS-PAGE staining with Coomassie blue R250. Finally, the Bradford assay was used to determine protein concentration^48^.

### Effect of AG1 on enzyme activity of G6PD variants

To determine the effect of AG1 on the activity of G6PD variants, the purified enzyme was incubated with 10 or 100 μM AG1 activator (MedChemExpress, NJ, USA) at 4 °C for 1 h. G6PD activity was determined and expressed as a percentage of the activity of the enzyme incubated in the absence of AG1.

### Determination of steady-state kinetic parameters

The steady-state kinetic parameters of G6PD variants were determined to assess the effect of mutations on their catalytic activity. G6PD activity was determined in a 1 ml cuvette by monitoring NADP^+^ reduction at 340 nm and 25 °C using a UV–VIS spectrophotometer (Shimadzu, Kyoto, Japan). The standard reaction mixtures were made up of 20 mM Tris–HCl pH 8.0, 10 μM MgCl_2_, 100 μM NADP^+^ and 500 μM G6P. The reaction was initiated with the addition of the enzyme. To determine the *K*_m_ for G6P, concentrations of G6P were varied from 5 to 500 μM while the concentration of NADP^+^ was held at 100 μM. The *K*_m_ for NADP^+^ was determined by varying the concentrations of NADP^+^ from 0.5 – 200 μM while keeping the concentration of G6P at 500 μM. *K*_m_, *k*_cat_ and *V*_max_ steady-state kinetic parameters were calculated by fitting the collected data to the Michaelis–Menten equation using GraphPad Prism software.

### CD analysis

To assess the effect of mutations on the secondary structure of G6PD variants, the secondary structure of the G6PD variants was evaluated spectroscopically by CD as previously described^21,23^. Before analysis, NaCl and imidazole were removed using Amicon® Ultra Centrifugal filters (Merck, Darmstadt, Germany). Far UV-CD spectra of the G6PD variants (0.15 mg/mL) were recorded in a 1 mm path-length quartz cuvette at 25 °C using a Jasco spectrometer, model J-815, equipped with a Peltier temperature control system. The CD spectra were collected at a scan rate of 50 nm/min over a wavelength range of 190–260 nm. For each sample, five scans were averaged, and the results of the buffer scans were subtracted.

### Structural analysis using intrinsic fluorescence and ANS-binding assays

Human G6PD contains seven tryptophan residues, which can be used to investigate changes in the 3D structure of G6PD variants. The intrinsic fluorescence emission spectra of the G6PD proteins (0.1 mg/mL) were monitored in a 96-well plate at 25 °C using a Synergy H1 hybrid reader (BioTek, VT, USA). The excitation wavelength was 295 nm and the emission spectra were recorded in the range of 300–400 nm. For ANS binding analysis, G6PD protein (0.1 mg/mL) was incubated with ANS (100 μM) for 1 h at 25 °C before measuring emission spectra between 400 and 600 nm with an excitation wavelength of 395 nm.

### Assessment of structural stability of G6PD variants

#### Thermal stability

To investigate the effect of G6PD variants on structural stability, thermal stability analysis was performed in a 20 μl reaction mixture, containing purified enzyme at a concentration of 0.2 mg/mL mixed with 5 × SYPRO™ Orange Protein Gel Stain (Thermo Fisher Scientific, San Jose, CA, USA). NADP^+^ was known to stabilize G6PD protein structure and AG1 was reported to promote dimerization^28,33^. Therefore, various concentrations of NADP^+^ (0, 10 and 100 μM) and AG1 activator (10 and 100 μM) were included in the reaction mixtures to assess whether these chemicals could improve protein stability. The reaction mixtures were heated at temperatures ranging from 20 to 80 °C in a LightCycler 480 real-time PCR machine (Roche, Mannheim, Germany) with excitation and emission wavelengths of 465 nm and 580 nm, respectively. The melting temperature (*T*_m_) was calculated for each G6PD variant and was defined as the temperature at which half of the protein unfolded.

#### Thermal inactivation

To assess the effect of mutation on the stability of G6PD variants, the enzyme was incubated for 20 min at temperatures ranging from 25 to 65 °C with varying concentrations of NADP^+^ (0, 10, and 100 μM) or AG1 activator (10 and 100 μM) before being cooled to 4 °C in a Thermocycler (Eppendorf, Hamburg, Germany). The residual enzyme activity was calculated as a percentage of the activity of the same enzyme incubated at 25 °C.

#### Protein stability in the presence of Gdn-HCl

Protein unfolding can be used to investigate structural stability during chemical denaturation. The stability of G6PD variants was determined by incubating protein samples for 2 h at 37 °C with varying concentrations of NADP^+^ (0, 10, and 100 μM) or AG1 activator (10 and 100 μM) in the presence of varying concentrations of Gdn-HCl (0.05, 0.1, 0.15, 0.2, 0.25, 0.3, 0.4, and 0.5 M). Following that, residual enzyme activity was determined and expressed as a percentage of the activity of the same enzyme incubated at 37 °C without Gdn-HCl.

#### Susceptibility of G6PD variants to trypsin digestion

To further evaluate the structural stability of G6PD variants, the susceptibility of G6PD proteins to trypsin was determined in the presence of different NADP^+^ concentrations (0, 10, and 100 μM) or AG1 activator (10 and 100 μM). Trypsin (final concentration of 0.5 mg/ml or 1250 U/ml) was incubated with recombinant G6PD protein for 5 min at 25 °C. Residual enzyme activity was determined and expressed as a percentage of the enzyme activity in the absence of trypsin.

### Determination of oligomeric state of G6PD variants

Size exclusion chromatography was used to determine the oligomeric state of the G6PD variants using AKTA fast protein liquid chromatography (FPLC) (GE Healthcare, Chicago, IL, USA) equipped with the Superdex 200 Increase 10/300 column. To monitor the effect of AG1 on promoting dimerization, the protein was incubated in the absence and presence of 500 μM AG1. Then, the recombinant protein (100 μg) was loaded onto the pre-equilibrated column. The chromatography was carried out at a flow rate of 0.5 ml/min with 50 mM Tris–HCl pH 7.5 and 150 mM NaCl. Blue dextran (> 2000 kDa), ferritin (440 kDa), catalase (232 kDa), aldolase (158 kDa), ovalbumin (43 kDa), chymotrypsinogen (25 kDa), and RNase A (13.7 kDa) were used to calibrate the column.

## Supplementary Information

Supplementary Information.

## References

[1] Eggleston LV, Krebs HA (1974). Regulation of the pentose phosphate cycle. Biochem. J..

[2] Luzzatto L, Ally M, Notaro R (2020). Glucose-6-phosphate dehydrogenase deficiency. Blood.

[3] Hirono A (1995). Molecular analysis of glucose-6-phosphate dehydrogenase variants in the Solomon Islands. Am. J. Hum. Genet..

[4] Vulliamy TJ (1988). Diverse point mutations in the human glucose-6-phosphate dehydrogenase gene cause enzyme deficiency and mild or severe hemolytic anemia. Proc. Natl. Acad. Sci. U S A.

[5] Hirono A, Beutler E (1988). Molecular cloning and nucleotide sequence of cDNA for human glucose-6-phosphate dehydrogenase variant A(-). Proc. Natl. Acad. Sci. U S A.

[6] Maeda M, Constantoulakis P, Chen CS, Stamatoyannopoulos G, Yoshida A (1992). Molecular abnormalities of a human glucose-6-phosphate dehydrogenase variant associated with undetectable enzyme activity and immunologically cross-reacting material. Am. J. Hum. Genet..

[7] Nantakomol D (2013). Evaluation of the phenotypic test and genetic analysis in the detection of glucose-6-phosphate dehydrogenase deficiency. Malar J..

[8] Li Q (2015). Prevalence and molecular characterization of glucose-6-phosphate dehydrogenase deficiency at the China-Myanmar border. PLoS ONE.

[9] Yan JB (2010). Rapid and reliable detection of glucose-6-phosphate dehydrogenase (G6PD) gene mutations in Han Chinese using high-resolution melting analysis. J. Mol. Diagn..

[10] Bancone G (2017). Prevalences of inherited red blood cell disorders in pregnant women of different ethnicities living along the Thailand-Myanmar border. Wellcome Open Res..

[11] Boonyuen U (2021). Glucose-6-phosphate dehydrogenase mutations in malaria endemic area of Thailand by multiplexed high-resolution melting curve analysis. Malar J..

[12] Gomez-Manzo S (2016). Glucose-6-phosphate dehydrogenase: update and analysis of new mutations around the world. Int. J. Mol. Sci..

[13] Beutler E (2008). Glucose-6-phosphate dehydrogenase deficiency: a historical perspective. Blood.

[14] Chu CS (2017). Haemolysis in G6PD heterozygous females treated with primaquine for *Plasmodium vivax* malaria: a nested cohort in a trial of radical curative regimens. PLoS Med..

[15] Chu CS, Bancone G, Nosten F, White NJ, Luzzatto L (2018). Primaquine-induced haemolysis in females heterozygous for G6PD deficiency. Malar J..

[16] Commons RJ, McCarthy JS, Price RN (2020). Tafenoquine for the radical cure and prevention of malaria: the importance of testing for G6PD deficiency. Med. J. Aust..

[17] Rueangweerayut R (2017). Hemolytic potential of tafenoquine in female volunteers heterozygous for glucose-6-phosphate dehydrogenase (G6PD) deficiency (G6PD Mahidol Variant) versus G6PD-normal volunteers. Am. J. Trop. Med. Hyg..

[18] Howes RE, Battle KE, Satyagraha AW, Baird JK, Hay SI (2013). G6PD deficiency: global distribution, genetic variants and primaquine therapy. Adv. Parasitol..

[19] Howes RE (2012). G6PD deficiency prevalence and estimates of affected populations in malaria endemic countries: a geostatistical model-based map. PLoS Med..

[20] Yoshida A, Beutler E, Motulsky AG (1971). Human glucose-6-phosphate dehydrogenase variants. Bull. World Health Organ..

[21] Boonyuen U (2017). A trade off between catalytic activity and protein stability determines the clinical manifestations of glucose-6-phosphate dehydrogenase (G6PD) deficiency. Int. J. Biol. Macromol..

[22] Boonyuen U (2016). Detailed functional analysis of two clinical glucose-6-phosphate dehydrogenase (G6PD) variants, G6PDViangchan and G6PDViangchan+Mahidol: decreased stability and catalytic efficiency contribute to the clinical phenotype. Mol. Genet. Metab..

[23] Praoparotai A, Junkree T, Imwong M, Boonyuen U (2020). Functional and structural analysis of double and triple mutants reveals the contribution of protein instability to clinical manifestations of G6PD variants. Int. J. Biol. Macromol..

[24] Wang XT, Lam VM, Engel PC (2005). Marked decrease in specific activity contributes to disease phenotype in two human glucose 6-phosphate dehydrogenase mutants, G6PD(Union) and G6PD(Andalus). Hum. Mutat..

[25] Gomez-Gallego F, Garrido-Pertierra A, Bautista JM (2000). Structural defects underlying protein dysfunction in human glucose-6-phosphate dehydrogenase A(−) deficiency. J. Biol. Chem..

[26] Gomez-Manzo S (2014). The stability of G6PD is affected by mutations with different clinical phenotypes. Int. J. Mol. Sci..

[27] Wang XT, Lam VM, Engel PC (2006). Functional properties of two mutants of human glucose 6-phosphate dehydrogenase, R393G and R393H, corresponding to the clinical variants G6PD Wisconsin and Nashville. Biochim. Biophys. Acta.

[28] Au SW, Gover S, Lam VM, Adams MJ (2000). Human glucose-6-phosphate dehydrogenase: the crystal structure reveals a structural NADP(^+^) molecule and provides insights into enzyme deficiency. Structure.

[29] Vlachos A, Westwood B, Lipton JM, Beutler E (1998). G6PD Mount Sinai: a new severe hemolytic variant characterized by dual mutations at nucleotides 376G and 1159T (N126D). Hum. Mutat..

[30] Beutler E, Kuhl W, Vives-Corrons JL, Prchal JT (1989). Molecular heterogeneity of glucose-6-phosphate dehydrogenase A. Blood.

[31] Nuchprayoon I, Louicharoen C, Charoenvej W (2008). Glucose-6-phosphate dehydrogenase mutations in Mon and Burmese of southern Myanmar. J. Hum. Genet..

[32] Nuchprayoon I, Sanpavat S, Nuchprayoon S (2002). Glucose-6-phosphate dehydrogenase (G6PD) mutations in Thailand: G6PD Viangchan (871G > A) is the most common deficiency variant in the Thai population. Hum. Mutat..

[33] Hwang S (2018). Correcting glucose-6-phosphate dehydrogenase deficiency with a small-molecule activator. Nat. Commun..

[34] Minucci A (2012). Glucose-6-phosphate dehydrogenase (G6PD) mutations database: review of the “old” and update of the new mutations. Blood Cells Mol. Dis..

[35] Horikoshi N (2021). Long-range structural defects by pathogenic mutations in most severe glucose-6-phosphate dehydrogenase deficiency. Proc. Natl. Acad. Sci. U S A.

[36] Kotaka M (2005). Structural studies of glucose-6-phosphate and NADP^+^ binding to human glucose-6-phosphate dehydrogenase. Acta Crystallogr. D Biol. Crystallogr..

[37] Bancone G (2017). The G6PD flow-cytometric assay is a reliable tool for diagnosis of G6PD deficiency in women and anaemic subjects. Sci. Rep..

[38] Phompradit P (2011). Prevalence and distribution of glucose-6-phosphate dehydrogenase (G6PD) variants in Thai and Burmese populations in malaria endemic areas of Thailand. Malar J..

[39] Matsuoka H (2007). Seven different glucose-6-phosphate dehydrogenase variants including a new variant distributed in Lam Dong Province in southern Vietnam. Acta Med. Okayama.

[40] Yan T (2006). Incidence and complete molecular characterization of glucose-6-phosphate dehydrogenase deficiency in the Guangxi Zhuang autonomous region of southern China: description of four novel mutations. Haematologica.

[41] He Y (2020). Glucose-6-phosphate dehydrogenase deficiency in the Han Chinese population: molecular characterization and genotype-phenotype association throughout an activity distribution. Sci. Rep..

[42] Jiang W (2006). Structure and function of glucose-6-phosphate dehydrogenase-deficient variants in Chinese population. Hum. Genet..

[43] Crooks RO, Rao T, Mason JM (2011). Truncation, randomization, and selection: generation of a reduced length c-Jun antagonist that retains high interaction stability. J. Biol. Chem..

[44] Cunningham AD, Colavin A, Huang KC, Mochly-Rosen D (2017). Coupling between protein stability and catalytic activity determines pathogenicity of G6PD variants. Cell Rep..

[45] Au SW (1999). Solution of the structure of tetrameric human glucose 6-phosphate dehydrogenase by molecular replacement. Acta Crystallogr. D Biol. Crystallogr..

[46] Wang XT, Chan TF, Lam VM, Engel PC (2008). What is the role of the second “structural” NADP^+^-binding site in human glucose 6-phosphate dehydrogenase?. Protein Sci..

[47] Raub AG (2019). Small-molecule activators of glucose-6-phosphate dehydrogenase (G6PD) bridging the dimer interface. ChemMedChem.

[48] Bradford MM (1976). A rapid and sensitive method for the quantitation of microgram quantities of protein utilizing the principle of protein-dye binding. Anal. Biochem..

