# Supplementary material for: Combined effects of double mutations on catalytic activity and structural stability contribute to clinical manifestations of glucose-6-phosphate dehydrogenase deficiency
Source: Sci Rep. 2021 Dec 21;11:24307. doi: 10.1038/s41598-021-03800-z (PMC8692357; doi:10.1038/s41598-021-03800-z)

Supplementary information

**Combined effects of double mutations on catalytic activity and structural stability contribute to clinical manifestations of glucose-6-phosphate dehydrogenase deficiency**

Phonchanan Pakparnich^1^, Sirapapha Sudsumrit^1^, Mallika Imwong^1^, Teeraporn Suteewong^2^, Kamonwan Chamchoy^3^, Danaya Pakotiprapha^4^, Ubolsree Leartsakulpanich^5^, Usa Boonyuen^1^*

^1^Department of Molecular Tropical Medicine and Genetics, Faculty of Tropical Medicine, Mahidol University, Bangkok, 10400, Thailand

^2^Department of Chemical Engineering, School of Engineering, King Mongkut’s Institute of Technology Ladkrabang, Bangkok, 10520, Thailand

^3^ Princess Srisavangavadhana College of Medicine, Chulabhorn Royal Academy, Bangkok 10210, Thailand

^4^Department of Biochemistry, Faculty of Science, Mahidol University, Bangkok, 10400, Thailand; Center for Excellence in Protein and Enzyme Technology, Faculty of Science, Mahidol University, Bangkok, 10400, Thailand

^5^National Center for Genetic Engineering and Biotechnology, National Science and Technology Development Agency, Pathumthani, 12120, Thailand

*Corresponding author: Usa Boonyuen

Department of Molecular Tropical Medicine and Genetics, Faculty of Tropical Medicine, Mahidol University, Bangkok, 10400, Thailand

Email: usa.boo@mahidol.edu

**Table S1**. Effect of AG1 on the activity of recombinant human G6PD variants.

| Construct | No treatment | 10 μM AG1 | 100 μM AG1 |
| --- | --- | --- | --- |
| WT | 100 ± 15 | 95 ± 7 | 109 ± 15 |
| Gaohe | 100 ± 22 | 110 ± 11 | 113 ± 5 |
| Mahidol | 100 ± 2 | 96 ± 5 | 83 ± 7 |
| Shoklo | 100 ± 9 | 104 ± 5 | 95 ± 6 |
| Canton | 100 ± 7 | 102 ± 14 | 101 ± 9 |
| Kaiping | 100 ± 10 | 108 ± 4 | 85 ± 11 |
| Gaohe + Kaiping | 100 ± 1 | 109 ± 5 | 105 ± 4 |
| Mahidol + Canton | 100 ± 11 | 110 ± 14 | 114 ± 7 |
| Mahidol + Kaiping | 100 ± 12 | 108 ± 2 | 102 ± 5 |
| Canton + Kaiping | 100 ± 8 | 83 ± 8 | 79 ± 19 |

**Table S2**. *T_m_* values from the thermal stability analysis of recombinant G6PD variants.

| Construct | T_m_ (°C) | | | | |
| --- | --- | --- | --- | --- | --- |
|  | Without ligand | 10 μM NADP^+^ | 100 μM NADP^+^ | 10 μM AG1 | 100 μM AG1 |
| WT | 55.08 ± 0.02 | 58.72 ± 0.01 | 61.88 ± 0.01 | 55.29 ± 0.02 | 58.59 ± 0.02 |
| Gaohe | 51.34 ± 0.02 | 56.67 ± 0.04 | 60.12 ± 0.05 | 51.22 ± 0.03 | 50.68 ± 0.02 |
| Mahidol | 51.65 ± 0.02 | 56.55 ± 0.04 | 60.74 ± 0.03 | 52.03 ± 0.02 | 50.54 ± 0.01 |
| Shoklo | 51.22 ± 0.02 | 55.15 ± 0.02 | 59.14 ± 0.02 | 51.10 ± 0.01 | 49.55 ± 0.02 |
| Canton | 48.36 ± 0.01 | 52.01 ± 0.02 | 55.18 ± 0.02 | 48.28 ± 0.01 | 48.54 ± 0.01 |
| Kaiping | 54.44 ± 0.03 | 59.61 ± 0.03 | 64.08 ± 0.02 | 54.27 ± 0.03 | 55.08 ± 0.01 |
| Gaohe + Kaiping | 52.22 ± 0.01 | 57.89 ± 0.01 | 62.12 ± 0.01 | 51.03 ± 0.01 | 50.03 ± 0.01 |
| Mahidol + Canton | 45.73 ± 0.01 | 49.03 ± 0.02 | 51.05 ± 0.06 | 45.42 ± 0.01 | 45.77 ± 0.01 |
| Mahidol + Kaiping | 54.19 ± 0.01 | 58.81 ± 0.01 | 62.09 ± 0.01 | 53.63 ± 0.01 | 53.52 ± 0.01 |
| Canton + Kaiping | 52.07 ± 0.03 | 59.48 ± 0.01 | 63.11 ± 0.01 | 54.32 ± 0.01 | 53.85 ± 0.01 |

**Table S3**. *T_1/2_* values from thermal inactivation analysis of recombinant G6PD variants.

| Construct | T_1/2_ (°C) | | | | |
| --- | --- | --- | --- | --- | --- |
|  | Without ligand | 10 μM NADP^+^ | 100 μM NADP^+^ | 10 μM AG1 | 100 μM AG1 |
| WT | 51.18 ± 0.02 | 55.89 ± 0.01 | 58.5 ± 0.5 | 50.87 ± 0.01 | 44.1 ± 0.9 |
| Gaohe | 46.1 ± 0.8 | 52.41 ± 0.01 | 56.89 ± 0.01 | 47.0 ± 0.4 | 42.1 ± 0.2 |
| Mahidol | 47.8 ± 0.1 | 52.8 ± 0.2 | 57.0 ± 0.5 | 50.11 ± 0.01 | 41.8 ± 0.5 |
| Shoklo | 47.01 ± 0.04 | 48.5 ± 0.1 | 53.7 ± 0.1 | 46.8 ± 0.6 | 36.4 ± 0.8 |
| Canton | 40.4 ± 0.2 | 49.0 ± 0.2 | 53.4 ± 0.3 | 44.5 ± 0.1 | 37.0 ± 1.1 |
| Kaiping | 49.4 ± 0.1 | 51.9 ± 0.1 | 56.5 ± 0.3 | 47.6 ± 0.6 | 44.3 ± 0.5 |
| Gaohe + Kaiping | 43.9 ± 0.8 | 54.8 ± 0.4 | 59.1 ± 0.2 | 48.7 ± 0.3 | 43.5 ± 0.5 |
| Mahidol + Canton | 36.3 ± 0.3 | 46.6 ± 0.4 | 50.9 ± 0.3 | 36.6 ± 0.4 | 33.6 ± 0.3 |
| Mahidol + Kaiping | 44.4 ± 0.7 | 52.3 ± 0.5 | 56.8 ± 1.4 | 47.7 ± 0.1 | 38.5 ± 0.4 |
| Canton + Kaiping | 43.0 ± 0.9 | 52.0 ± 0.9 | 54.0 ± 2.1 | 49.9 ± 0.1 | 37.3 ± 1.3 |

**Table S4**. *C_1/2_* values from structural stability analysis in the presence of Gdn-HCl of recombinant G6PD variants.

| Construct | C_1/2_ (M) | | | | |
| --- | --- | --- | --- | --- | --- |
|  | Without ligand | 10 μM NADP^+^ | 100 μM NADP^+^ | 10 μM AG1 | 100 μM AG1 |
| WT | 0.240 ± 0.003 | 0.34 ± 0.01 | 0.844 ± 0.001 | 0.163 ± 0.003 | 0.103 ± 0.001 |
| Gaohe | 0.113 ± 0.002 | 0.218 ± 0.005 | 0.37 ± 0.02 | 0.112 ± 0.002 | 0.094 ± 0.001 |
| Mahidol | 0.12 ± 0.01 | 0.23 ± 0.01 | 0.29 ± 0.04 | 0.165 ± 0.002 | 0.112 ± 0.001 |
| Shoklo | 0.134 ± 0.004 | 0.211 ± 0.004 | 0.284 ± 0.006 | 0.089 ± 0.007 | 0.070 ± 0.002 |
| Canton | 0.043 ± 0.002 | 0.12 ± 0.01 | 0.19 ± 0.01 | 0.092 ± 0.003 | 0.043 ± 0.001 |
| Kaiping | 0.16 ± 0.01 | 0.24 ± 0.01 | 0.38 ± 0.01 | 0.12 ± 0.01 | 0.10 ± 0.01 |
| Gaohe + Kaiping | 0.11 ± 0.01 | 0.15 ± 0.01 | 0.24 ± 0.01 | 0.20 ± 0.01 | 0.11 ± 0.01 |
| Mahidol + Canton | 0.02 ± 0.01 | 0.16 ± 0.01 | 0.22 ± 0.01 | 0.01 ± 0.01 | <0.01 |
| Mahidol + Kaiping | 0.130 ± 0.001 | 0.246 ± 0.004 | 0.39 ± 0.02 | 0.161 ± 0.001 | 0.096 ± 0.001 |
| Canton + Kaiping | 0.06 ± 0.01 | 0.10 ± 0.02 | 0.11 ± 0.01 | 0.12 ± 0.01 | 0.10 ± 0.01 |

**Table S5**. Residual enzyme activity upon trypsin digestion of recombinant G6PD variants.

| Construct | % Residual enzyme activity | | | | |
| --- | --- | --- | --- | --- | --- |
|  | Without ligand | 10 μM NADP^+^ | 100 μM NADP^+^ | 10 μM AG1 | 100 μM AG1 |
| WT | 17 ± 4 | 59 ± 3 | 76 ± 10 | 18 ± 2 | 32 ± 5 |
| Gaohe | 11 ± 4 | 54 ± 6 | 63 ± 2 | 15 ± 4 | 17 ± 3 |
| Mahidol | 9 ± 2 | 58 ± 1 | 70 ± 4 | 18 ± 3 | 49 ± 4 |
| Shoklo | 1.6 ± 0.5 | 44 ± 2 | 55 ± 2 | 6.3 ± 0.7 | 7 ± 1 |
| Canton | 4.1 ± 0.1 | 36 ± 3 | 52 ± 11 | 11 ± 1 | 13 ± 1 |
| Kaiping | 39 ± 1 | 89 ± 16 | 96 ± 16 | 47.2 ± 0.2 | 57 ± 9 |
| Gaohe + Kaiping | 30 ± 3 | 70 ± 10 | 70 ± 13 | 34 ± 2 | 31 ± 5 |
| Mahidol + Canton | 3 ± 2 | 15 ± 1 | 29 ± 3 | 2 ± 1 | 5 ± 2 |
| Mahidol + Kaiping | 57 ± 3 | 90 ± 1 | 99 ± 1 | 56 ± 2 | 47 ± 2 |
| Canton + Kaiping | 27 ± 1 | 63 ± 1 | 73 ± 2 | 35 ± 3 | 37 ± 6 |

**Table S6**. Primers used for site-directed mutagenesis.

| **Primer** | **Sequence** |
| --- | --- |
| Gaohe_F | 5’-GTCGGATACAGGCATATTCATCA-3’ |
| Gaohe_R | 5’-TGATGAATATGCGTGTATCCGAC-3’ |
| Mahidol_F | 5’-CGAGTCCTGCATGAGCCAGATAAGCTGGAA-3’ |
| Mahidol_R | 5’-TTCCAGCTTATCTGGCTCATGCAGGACGACTCG-3’ |
| Shoklo_F | 5’-CGCCTGCGTTACCCTCACCTTCA-3’ |
| Shoklo_R | 5’-GAACGTGAGGGTAACGCAGGCGA-3’ |
| Canton_F | 5’-GACGAGCTCCTTGAGGCCTGG-3’ |
| Canton_R | 5’-CCAGGCCTCAAGGAGCTCGTC-3’ |
| Kaiping_F | 5’-GCCTGGCATATTTTCACCCC-3’ |
| Kaiping_R | 5’-GGGGTGAAAATATGCCAGGC-3’ |

**Figure S1**. Locations of the mutations in G6PD variants analyzed in this study, mapped on the 3D structure of the enzyme. (A) Ribbon and (B) surface presentation of monomeric human G6PD. Mutations are indicated in red, G6P is presented in yellow and NADP^+^ molecules are shown in purple.


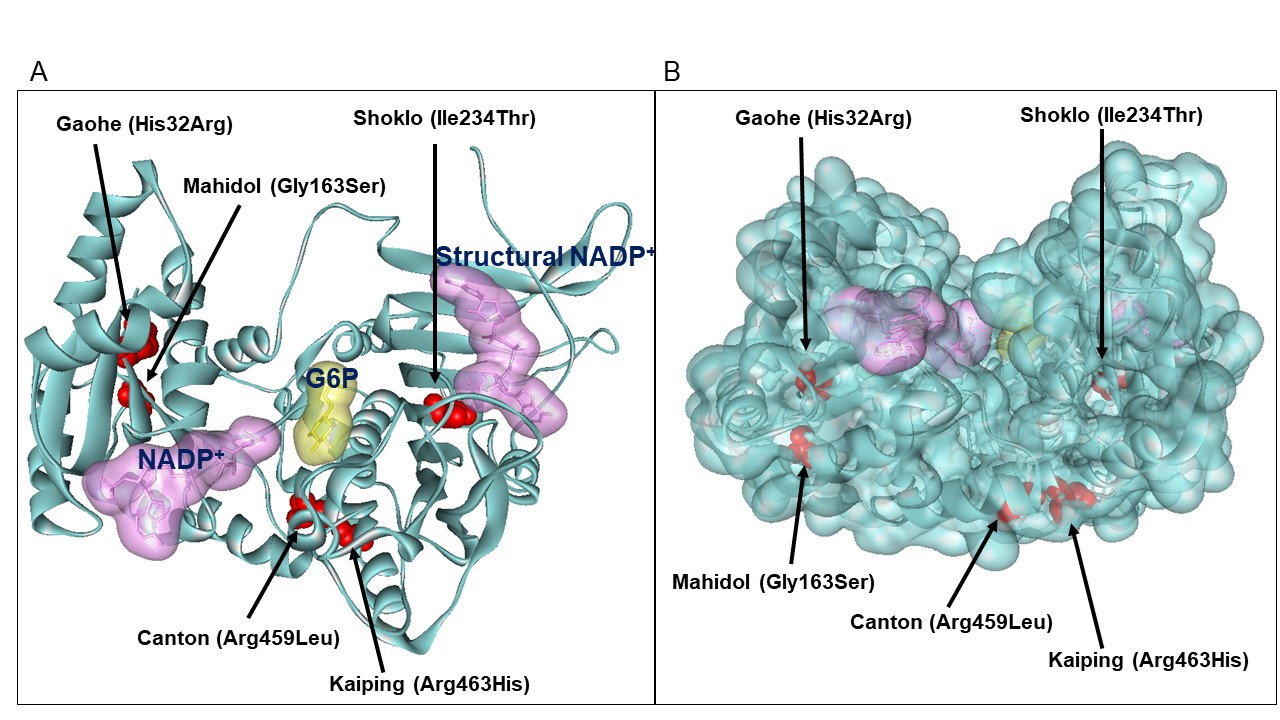

Supplement: Supplementary file 1 — Supplementary Information. [file 41598_2021_3800_MOESM1_ESM.docx]
